# The Impact of Modern Lifestyles on Spinal Health in the Pediatric Population: A Narrative Review

**DOI:** 10.3390/children13030341

**Published:** 2026-02-27

**Authors:** Katarzyna Zaborowska-Sapeta, Patrycja Tymińska-Wójcik, Anelise Sonza, Marek Kluszczyński, Agnieszka Skowrońska

**Affiliations:** 1 Department of Rehabilitation and Orthopaedics, School of Medicine, University of Warmia and Mazury in Olsztyn, 10-719 Olsztyn, Poland; 2 Regional Specialized Children’s Hospital in Olsztyn, 10-561 Olsztyn, Poland; 3 Department of Electrical Devices and High Voltage Technology, Lublin University of Technology, Nadbystrzycka 38A, 20-618 Lublin, Poland; p.tyminska@pollub.pl; 4 Department of Physical Therapy, Postgraduate Program in Human Movement Sciences, Santa Catarina State University, Florianopolis 88035-001, Santa Catarina, Brazil; anelise.sonza@udesc.br; 5 Department of Pediatric Neurology, Jagiellonian University, 31-007 Krakow, Poland; marek.kluszczynski@uj.edu.pl; 6 Pediatric Rehabilitation Department, University Children’s Hospital of Krakow, Wielicka 265, 30-663 Krakow, Poland; 7 Department of Human Physiology and Pathophysiology, School of Medicine, University of Warmia and Mazury in Olsztyn, 10-082 Olsztyn, Poland; agnieszka.skowronska@uwm.edu.pl

**Keywords:** lifestyle, adolescence, screen time, spine development

## Abstract

**Highlights:**

**What are the main findings?**

- Until now, discopathy was believed to be rare in children and adolescents and to result mainly from post-traumatic conditions. Meanwhile, changing lifestyles among the youngest generation are causing significant shifts in the epidemiology and clinical presentation of patients with early spinal dysfunction.

**What are the implications of the main findings?**

- Identifying modifiable factors that impede spinal growth could help prevent early dysfunction, which is crucial to the correct development of the younger generation.

**Abstract:**

**Background:** Children’s behavior and lifestyle are changing rapidly, potentially exceeding the capacity of physiological adaptation. Contemporary lifestyles may negatively affect spinal development and contribute to dysfunction and premature degeneration. Despite the increasing prevalence of postural changes, cervical spine disorders in adolescents remain under-researched. **Methods:** This narrative review is based on a comprehensive search of PubMed/MEDLINE and Scopus. The search strategy included a broad review of anatomical and biomechanical literature from the past 25 years and a focused review of studies from the last 15 years to reflect recent generational changes. **Results:** The immature spine has distinct structural and biomechanical characteristics that increase susceptibility to maladaptive responses to unbalanced forces. High screen time is associated with sedentary behavior and increased consumption of ultra-processed foods, which may affect metabolic health and musculoskeletal development. Childhood and adolescent obesity are increasingly prevalent and may influence spinal development, including through myosteatosis. Data on the consequences of cervical and lumbar lordosis loss in adolescents remain limited. Although degenerative spinal disorders are well recognized in adults, their identification in younger populations may be inadequate. **Conclusions:** Modern lifestyle factors pose a growing risk to children’s spinal health through complex interactions among behavioral, metabolic, and biomechanical mechanisms. The developing spine’s vulnerability and the coexistence of multiple, interrelated risk factors support the need for integrated preventive strategies rather than single-factor interventions. Future studies should focus on models capturing these interactions and their long-term consequences.

## 1. Introduction

In this review, we address concerns about the consequences of spinal maldevelopment during adolescence amid contemporary lifestyle changes. Prolonged sitting, sustained neck flexion, and static spinal loading during critical growth periods may influence spinal development and contribute to functional limitations in early adulthood. The spine is a complex, integrated structure that provides stability and strength while allowing mobility. Its growth and maturation extend over many years and continue until ossification is complete. Proper morphological development of the spinal column is essential for effective load distribution and long-term mechanical performance. Congenital or acquired disturbances in this process may increase susceptibility to degenerative changes and chronic pain syndromes later in life. Due to their distinct anatomical and biomechanical characteristics, the spines of children and adolescents are particularly sensitive to maladaptive or asymmetric mechanical stress. Early and prolonged exposure to non-neutral postures, including extended use of mobile devices, may interfere with the development of normal physiological spinal curvatures [1]. Alterations in sagittal alignment can, in turn, reduce the spine’s capacity to tolerate mechanical loads. Clinical manifestations of these changes may not be immediately apparent, and the duration of an asymptomatic period can vary. Nevertheless, neurological impairment may develop and present with a range of symptoms. Importantly, studies that rely solely on pain complaints in children and adolescents may not adequately reflect the underlying pathological processes [2,3]. Spinal pain often represents the outcome of long-standing structural or functional alterations and, therefore, may be of limited value as a primary endpoint in younger populations. In recent years, the medical literature has increasingly reported a younger age at presentation among patients with spinal disorders [4,5,6,7]. This trend is accompanied by growing demand for specialist diagnostics, conservative treatment, and, in selected cases, surgical treatment among younger age groups [8]. The rising prevalence of these problems can place a significant burden on health systems, both economically and through long-term social consequences, underscoring the importance of preventive measures and early identification of factors that disrupt spinal development [9,10].

## 2. Methods

In preparation for this narrative review, a comprehensive search of the biomedical literature was conducted using PubMed/MEDLINE and Scopus. The initial database search was divided into two parts: first, a broad search covering 25 years related to anatomy and spine biomechanics; and second, a more recent search limited to the last 15 years to reflect ongoing processes. The search was limited to English-language papers, and selected keywords included: ‘adolescent and cervical spine’, ‘children and spine development’, ‘adolescent and screentime’, ‘forward head posture’, ‘high processed food and spine’, and ‘myosteatosis and spine’. Narrative synthesis was used to summarize findings on the current state of knowledge regarding biomechanical differences in the growing spine and potential factors that may adversely affect it and are associated with lifestyle patterns in children and adolescents.

## 3. Results

### 3.1. The Growing Spine—Anatomy and Biomechanics

The development of the spine begins in the third week of gestation. It undergoes a long process of growth, formation, maturation, chondrification, and ossification, all of which are precisely regulated by genes, mechanical forces, movement, hormones, nutrients, and time. The immature spine is a mosaic of primary growth centers composed of cartilaginous tissue responsible for longitudinal spinal growth (endochondral ossification) [11,12,13]. Ossification of the cartilaginous part of the vertebral endplate (CEP) begins around the age of five, leading to the formation of the ring apophysis (secondary ossification growth centres), which are essential for the structural maturation of the vertebral bodies. The ring apophysis should be fully formed by the age of 12, but fusion does not occur until skeletal maturity [14]. Spine maturation is a complex and highly individual process. The study by Costa et al. describes ring apophysis fusion patterns that vary by age, spinal level, and sex. According to this study, ossification of the ring apophysis occurs between the ages of 9 and 15 in males and 7 and 15 in females. Fusion occurs between the ages of 14 and 19 in both sexes [15]. Radiographic studies of the cervical spine revealed that the earliest apophysis was observed in a patient aged 3 years, and that all patients aged 14 years and older exhibited the apophysis. The inferior apophysis of a cervical vertebral body appeared before the superior apophysis; however, the superior apophysis fused first in most cases (86%). The superior apophyses have fused by the age of 18, although unfused inferior apophyses were still evident in the 20-year-old group [16]. All maturation processes aim to strengthen the spine’s resilience to the mechanical effects of growing body mass and control shifts in the body’s center of mass [14,17]. Due to its increased elasticity and enhanced resistance to compressive forces, the paediatric spine is less susceptible to fractures following low-energy trauma [17]. However, severe accidents often result in cervical spine injuries, which can lead to lifelong disability [18]. The biomechanics of the growing spine differ from those of the adult spine, and certain anatomical features make the former more susceptible to stress and load (Table 1).

### 3.2. Static Versus Dynamic Spinal Load

**Figure 1:**
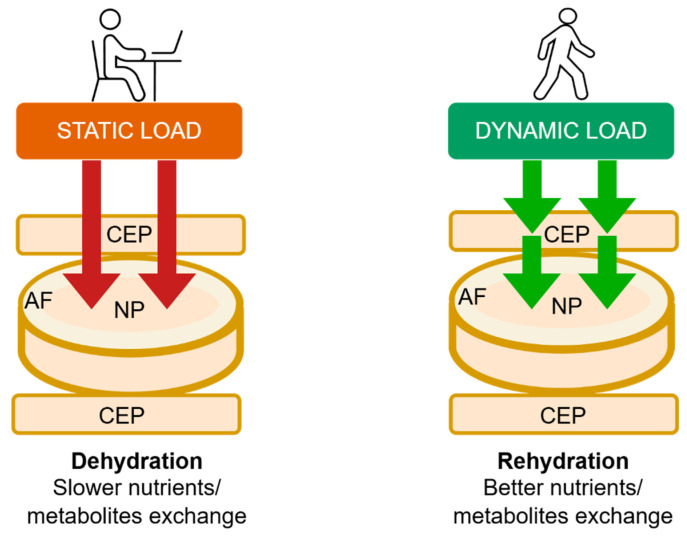
This diagram illustrates the differences between the effects of static and dynamic forces on the disc. The red arrows represent static loads, such as prolonged sitting, that cause disc dehydration and slow nutrient and metabolite diffusion, disrupting the internal homeostasis of the nucleus pulposus. The green arrows represent dynamic loads that enhance hydration and facilitate molecular diffusion. NP—Nucleus Pulposus, CEP—Cartilage Endplate, AF—Annulus Fibrosus. Source: prepared by the authors (2025).

Forces acting on the musculoskeletal system are crucial in shaping the growing spine. These forces, including compression, tension, shear, flexion-extension, and hydrostatic pressure, participate in biological regulatory pathways through mechanotransduction [19,20]. The intervertebral disc (IVD) plays a critical role in load transmission and flexibility, thereby contributing to the overall stability of the entire vertebral column. This stability is also influenced by the morphological properties of the vertebral bodies, including their shape, structural composition, and resistance to mechanical loading [21,22,23]. During maturation, the IVD gradually decreases in hydration and vascularization, and its cellular matrix composition changes [24]. A defining feature of the young spine is its high disc hydration, which increases during the first two decades of life, due to enhanced glycosaminoglycan synthesis in the nucleus pulposus (NP) [25,26]. Hydration and IVD osmolality are dynamic and influenced by applied mechanical load. The NP of the intervertebral disc is a highly permeable, gelatinous tissue that adapts to vertical stresses by regulating its water content. NP cells respond to osmotic fluctuations by regulating water flow through aquaporins, which are water channel proteins [27]. Previous studies have demonstrated the key roles of aquaporins 1 (AQP1) and 3 (AQP3) in nutrient delivery and metabolic substance transport in NP tissue. The expression of aquaporins in IVD cells is regulated by the osmotic environment; for example, a decrease in osmotic pressure reduces AQP1 expression. Notably, AQP1 also exhibits significant oxygen permeability. Under hypoxic conditions, AQP1 upregulation facilitates oxygen transport across membranes, potentially mitigating hypoxia-induced damage in the disc. Results show that as the degree of degeneration of human NP tissue increases, its water content decreases, leading to a progressive reduction in AQP levels [27]. CEPs play a crucial role in IVD development by maintaining metabolic homeostasis and supplying nutrients to the NP [21,28]. Disc nutrition depends on fluid flow within the NP, driven by a pressure gradient. Even under constant mechanical loading, significant pressure gradients persist within the NP, particularly during the initial loading phase and in more hydrated IVDs. Pressure peaks at the center of the nucleus and decreases axially toward the CEP and radially toward the annulus fibrosus (AF) [29]. As static loading and disc dehydration increase over time, circulation within the disc slows, as does the exchange of metabolites and nutrients through the CEP (Figure 1). Studies have shown that constant pressure gradually reduces the number of vessel buds in the CEP and significantly decreases vascular endothelial growth factor (VEGFA) concentrations [30]. Compared with constant static loading, dynamic mechanical forces enhance the diffusion and convection of essential nutrients within the IVD, such as oxygen and glucose, while also facilitating the removal of metabolic waste products. Cyclic mechanical loading favors the maintenance of a larger viable cell population and promotes extracellular matrix synthesis, thereby improving disc hydration and overall disc health [31,32]. The role of exercise in preventing degenerative disc disease remains a topic of debate. High physical strain and competitive sports can result in spinal pain and adverse changes [33]. Studies indicate that moderate effort and an upright, bipedal posture benefit IVD health [34,35,36].

### 3.3. The Importance of Sagittal Profile Development

**Figure 2:**
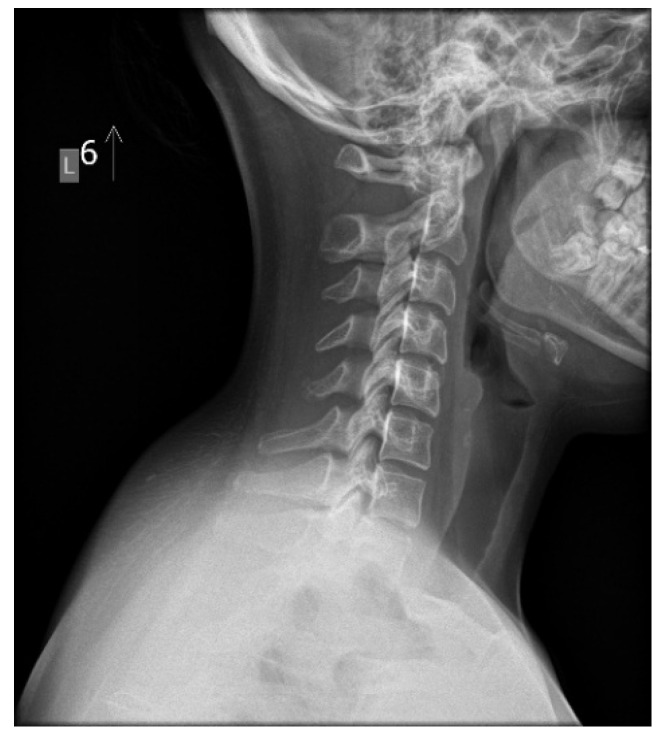
A symptomatic 17-year-old girl with military neck—chronic headaches, numbness, and weakness of the right hand. Source: File from an outpatient clinic (2025).

**Figure 3:**
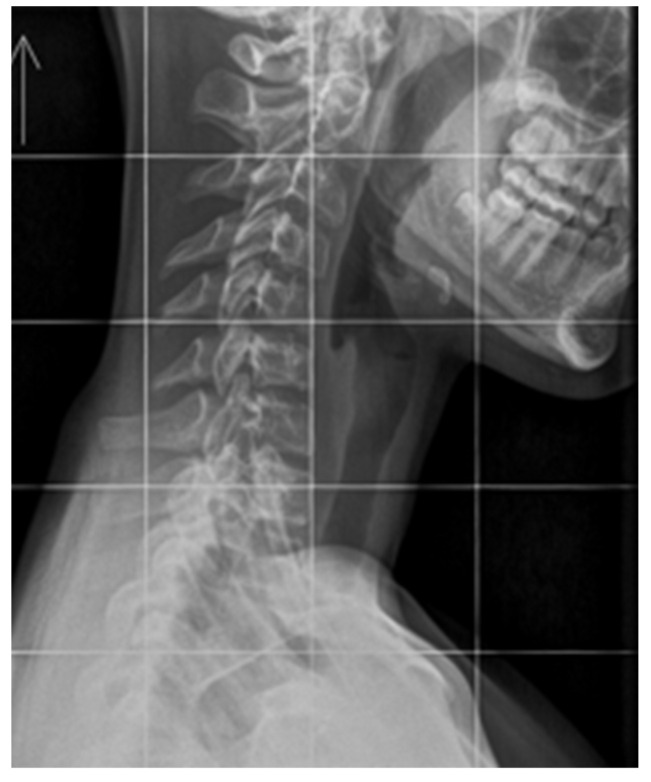
An asymptomatic 14-year-old girl with an inverted cervical spinal curve. Source: File from an outpatient clinic (2025).

Anatomically, the spine exhibits four natural curvatures in the sagittal plane: cervical lordosis, thoracic kyphosis, lumbar lordosis, and sacral kyphosis. These natural curvatures are evolutionary adaptations that enable the body to maintain an upright posture with minimal energy expenditure and maximal resistance to gravity [37,38]. Proper formation of these curvatures prevents excessive stress on intervertebral discs, ligaments, and muscles by efficiently distributing forces and transferring mechanical loads to each moving segment [39]. The biomechanical balance of the counter curves provides optimal stabilization of the entire spine. Disturbances to this mechanical system can contribute to a gradual and often prolonged process of tissue degeneration. Clinical symptoms appear when the compensation limit is reached and the critical point of permanent tissue damage is exceeded (Figure 2) [40]. The presence of abnormal spinal curvatures in the absence of clinical symptoms, such as pain, does not necessarily indicate a normal state, despite the surprisingly high prevalence of such cases (Figure 3). Lee et al. conducted a radiographic analysis of sagittal alignment in 181 asymptomatic adolescents. They found a high prevalence of cervical hypolordosis in 13–17-year-olds (71%) and 8–12-year-olds (65.6%). The overall prevalence of cervical kyphosis in the studied population was approximately 40% [41]. These findings provide valuable insight into the extent of maldevelopment of cervical lordosis in young populations. There is a clear need for further research focused specifically on the paediatric population, particularly studies examining the influence of lifestyle factors, such as prolonged static postures with sustained cervical flexion, as we commonly observe in children and adolescents when using mobile phones, tablets, and other screen-based devices. These studies should also encompass a broader range of symptoms other than just cervical pain, as this is not typical in children and adolescents. The spinal sagittal profile matures gradually during growth and development. Studies establishing reference values for sagittal curvatures in the growing spine are limited, particularly across different age groups and sexes. Research by Takács et al. suggests that relating normal physiological curvatures to height rather than age provides a more accurate assessment [42]. Multicenter studies involving large populations have demonstrated that changes in thoracic kyphosis and lumbar lordosis occur during growth spurts in children and are interdependent, forming a specific, cascading phenomenon [43]. Unfortunately, many valuable studies do not examine the cervical sagittal profile, an integral part of overall spinal alignment [43,44].

### 3.4. Adolescents, Technology, and Nomophobia

The time we spend in front of digital devices is increasing year after year, and each generation is becoming more dependent on the digital world. According to the Common Sense Census, 84% of teenagers own smartphones, and 70% use social media multiple times a day [35]. During the COVID-19 pandemic, the total daily screen time increased from 4 h and 44 min to 5 h and 33 min for tweens, and from 7 h and 22 min to 8 h and 39 min for teens [45]. This increase has been significantly faster over the past two years than over the previous four [45]. A study of 384,591 15-year-olds from 55 countries found that children start using digital devices at a very early age. The results showed that 7.53% start before the age of three, 27.83% start between the ages of four and six, 35.56% start at the age of seven or nine, 21.07% start at the age of ten or twelve, and only 6.85% after the age of thirteen [46]. These results indicate that 70.92% of children begin using digital devices before age 10. A study of 237 children aged 3 to 5 years who averaged nearly 4 h of screen time per day found a positive association between parental and child screen use, indicating that familial habits and behaviors significantly influence screen exposure in young children [47]. Hantal et al. conducted a study linking discopathy in adolescents to screen time. The study involved 94 patients with disc pathology (bulging, protrusion, extrusion) aged 10–16 who spent an average of 5.16 h per day on digital devices. This was also associated with low physical activity levels [48]. This is the first and most significant study to highlight the impact of adolescents’ lifestyles on early spine degeneration. Smartphone addiction is a significant challenge in today’s society. The term nomophobia (no mobile phone phobia) was introduced into scientific literature with the rise in excessive phone use. This refers to the fear of losing access to one’s phone, its content, and connectivity with a particular community [49]. A meta-analysis of 52 studies involving almost 47,400 participants from 20 countries found that the overall prevalence of nomophobia across all symptom and severity categories was 93.92%. Mild nomophobia symptoms were observed in around 25.80% of individuals, moderate symptoms in approximately 52.40%, and severe symptoms in around 20.35% [50].

Excessive or problematic screen media use can be examined with respect to various health outcomes, but psychological hazards are the main focus [51]. We believe that insufficient research has been conducted on the impact of early and long-term use of digital devices by children and adolescents on the development of the skeletal system, including posture and spinal development. This can have negative long-term health consequences [52,53]. Spending extended periods sitting in front of digital media also contributes to a sedentary lifestyle, which has implications for musculoskeletal development, including low physical activity [54,55,56,57]. Trends in insufficient physical activity among adolescents were examined between 2001 and 2016. Data from 298 surveys in 146 countries, covering 1.6 million students, indicated that more than 80% of adolescents did not meet the WHO’s physical activity recommendations. This study highlights the importance of interventions to promote physical activity, particularly among girls [58]. A cohort study of children aged 11 to 12 found that the more time spent in front of a screen, the greater the risk of experiencing moderate or severe spinal pain. Interestingly, this link was independent of physical activity levels [59], which may be explained by the effects of constant static forces on the spine and muscles. Spending extended hours using digital media replaces an active lifestyle, raising the risk of pain and spinal maldevelopment issues even further [60]. To synthesize the available epidemiological and clinical evidence linking screen exposure and sedentary behaviors with musculoskeletal outcomes in children and adolescents, the key studies are summarized in Table 2.

### 3.5. Forward Head Posture Syndrome and Cervicovagopathy

This syndrome is characterized by stress on the cervical spine tissues caused by prolonged flexion resulting from mobile device use. Several terms describing the same anatomical and pathological issue of forward head protraction appear in the literature, including text neck syndrome, tech neck syndrome, turtleneck syndrome, and forward head posture syndrome [60,61,62,63,64]. Earlier generations of mobile phones were primarily used for communication, and users frequently bent over to text, which led to the development of text neck syndrome. Today, smartphones offer a wider range of functions, and young people often use them for hours at a time to watch films, play games, and chat with friends. In broader terms, concepts less affected by technological change tend to be more universal. Forward head posture (FHP) refers to the position of the head and neck, regardless of the activity being performed.

The cervical spine supports the weight of the head while enabling multi-directional movement. This is achieved through the deep muscles of the cervical spine, which aid in perception and stabilize posture, along with the vestibular system and visual input. The upper cervical spine, particularly C1–C3, and the altantooccipital junction are particularly important. It is estimated that the suboccipital muscles contain a high number of spindles (proprioceptors) [65]. The activity of these mechanoreceptors can be affected by injury, inflammation, or degenerative changes [66]. Loss of cervical lordosis is a structural disorder that can be observed on neck X-rays in a relaxed, habitual posture (Figure 2 and Figure 3). Muscles have a high capacity to adapt to altered working conditions in non-physiological positions by shortening, atrophy, and degenerative changes. This can impair muscle strength and endurance, creating a vicious cycle whereby incorrect posture leads to changes in muscle morphology and innervation. These changes then cause weakened strength and neurocontrol, which further deteriorate stability and posture. These morphological changes in muscles also cause abnormal, continuous sensory impulses from the paraspinal and suboccipital muscles (deep stabilizers) to the central nervous system, which is responsible for posture, balance, and coordination, resulting in information noise [66,67]. Studies have demonstrated a direct correlation between these morphological alterations and symptoms, such as chronic headaches, neck pain, somatic dysfunction, and balance disorders [68,69]. The clinical signs of FHPS reported in the literature are diverse and often nonspecific. These include pain and limited cervical spine mobility, increased neck muscle tension, headaches, dizziness, balance problems, visual disturbances, and fatigue. Less common clinical issues associated with FHPS include chest pain, brain fog, postural orthostatic tachycardia syndrome, sleep disturbances, gastrointestinal problems such as gastroparesis, and tinnitus [67,68,69,70,71]. The variety and nonspecific nature of symptoms associated with FHPS can be attributed to cervicovagopathy, a term and new hypothesis introduced by Hauser [70,71]. The vagus nerve (VN) is the longest cranial nerve and carries sensory, motor, and parasympathetic fibers to organs in the chest and abdomen. Anatomically, the VN is closely associated with the C1 and C2 vertebrae. The superior vagal ganglion is located in the lateral part of the jugular foramen, and the inferior ganglion is situated below and in front of the transverse processes of the C1 and C2 vertebrae, where compression and/or stretching of the nerve structures most often occur. Abnormal spinal anatomy or slight instability of the upper cervical spine can cause conflict between the vertebrae and the vagus nerve and/or its ganglia (cervicovagopathy). Due to the complex functions of the X nerve (autonomic, sensory, and motor), its dysfunction can manifest as diverse symptoms affecting many organs and systems [70,71]. It should be emphasized that cervicovagopathy is a novel hypothesis that requires further research and clinical observation to substantiate. Although the proposed pathophysiological mechanisms are comprehensive and logical from anatomical and neurological perspectives, they require confirmation in prospective clinical studies with a control group to establish a cause-and-effect relationship between cervical spine changes and autonomic dysfunction. The cervicovagopathy hypothesis is based primarily on clinical observations, imaging studies, and therapeutic outcomes in adults. Despite these limitations, the concept of cervicovagopathy deserves attention for its complexity and potential clinical implications, especially given the rising prevalence of FHPS in children and adolescents.

### 3.6. Highly Processed Food Consumption, Obesity, and Myosteatosis

Proper nutrition and physical activity are essential for a child’s growth and development. However, spending many hours in the digital world is often associated with increased snacking and the replacement of full meals with ultra-processed foods (UPF). This trend is driven by the widespread availability and easy preparation of these products [72,73]. Unfortunately, analyses of evolving dietary habits show that UPF consumption is increasing among young people [74,75]. There is a strong connection between prolonged screen time and UPF consumption. A cross-sectional study of four-year-old children (n = 362) found that screen time exceeding 60 min was associated with lower developmental scores, greater screen-based eating, and higher UPF consumption [76]. Studies among adolescents suggest that sedentary behaviours associated with screen time may lead to impulsive eating and a preference for convenience foods, which are often UPF [77]. These findings could also pose risks to the proper development of the adolescent skeletal system tissues. The impact of UPF on children’s health is now being discussed not only among specialists but also within highly developed societies, where the adverse health effects of high consumption are evident. The impact of UPF on skeletal and muscular development remains unclear. An experimental study in rats showed that UPF can disrupt endochondral ossification in the skeleton. Young rats fed UPF exhibited skeletal pathologies, including growth retardation, decreased bone mineral density, structural deterioration, and poor skeletal tissue quality [78]. Studies on mice have shown that UPF negatively affects bone quality, increases marrow fat, alters growth patterns, and modifies the gut microbiome [79]. UPFs also harm muscle mass. Studies in adults have revealed a significant correlation between increased UPF intake and a higher risk of reduced muscle mass and grip strength [80,81]. An inadequate diet is a well-known risk for developing metabolic syndrome (MS), which is an increasing concern in the pediatric population [82]. The effect of metabolic disorders on osteoarthritis and intervertebral disc diseases (IVDD) development has been widely studied, primarily in adults. A study involving 928 participants showed that individuals with one or more components of MS had a higher risk of IVDD compared to those without MS components, suggesting that the accumulation of metabolic abnormalities increases the likelihood of IVDD [83]. Hyperglycemia and dyslipidemia are also recognised as risk factors for IVDD severity [84,85]. A significant limitation of these studies is that they have been conducted in adults. The results should not be directly extrapolated to the pediatric population. Obesity in children and adolescents is an increasingly prevalent issue with complex effects on health and development. When considering how lifestyle influences the musculoskeletal system, obesity should not be overlooked. In children, obesity affects musculoskeletal development through changes in hormonal environments, including shifts in growth and pubertal hormones, as well as alterations in adipokine release. Furthermore, different fat compartments, particularly visceral fat, can negatively impact bone quality and strength [86,87]. Obesity has also been linked to myosteatosis, characterized by increased fat infiltration of muscles and adverse effects on strength and metabolic function [88,89].

## 4. Limitations

The interpretation of the available literature is limited by several methodological challenges. Lifestyle is a multidimensional construct in which screen time, physical activity, and diet represent interdependent behaviors. Children and adolescents with prolonged screen exposure often present with low physical activity and inadequate nutrition, both of which may independently influence spinal health. Isolating the effects of single factors remains difficult, especially when additional variables such as genetics, socioeconomic status, and environment are considered. A further challenge stems from the heterogeneity of methods and definitions used across studies. Terms such as excessive screen time, loss of cervical lordosis, or forward head posture syndrome are often defined differently, preventing direct comparisons and meta-analytical synthesis. Moreover, the limited availability of prospective imaging studies in pediatric populations—primarily due to ethical restrictions and concerns about radiation exposure—hinders our understanding of the natural course of spinal changes during growth. As most evidence is derived from retrospective or short-term data, the prognostic relevance of early structural abnormalities remains uncertain. Finally, the predominance of cross-sectional designs precludes establishing causality. It remains unclear whether spinal pain triggers reduced activity and increased sedentary time, or whether these lifestyle factors precede and contribute to spinal pathology.

## 5. Strengths

Despite these limitations, the review offers several strengths. It provides a multidisciplinary synthesis that integrates anatomical, biomechanical, radiological, pediatric, nutritional, and public health perspectives—an approach essential for understanding the complex determinants of spinal health in youth. By emphasizing the developmental specificity of the growing spine, the review highlights its greater susceptibility to lifestyle-related stressors than that of adults. Finally, the focus on modifiable factors underscores practical implications for prevention and health promotion. Behavioral, educational, and environmental interventions targeting current generational habits have the potential to mitigate future spinal dysfunction and improve well-being.

## 6. Conclusions

Contemporary lifestyle patterns among children and adolescents constitute a growing public health concern. The interplay of behavioral, metabolic, and biomechanical factors appears to accelerate the onset of spinal dysfunction and early degenerative changes. The pediatric spine, characterized by its dynamic anatomy and ongoing growth, is particularly sensitive to environmental and behavioral influences.

Based on the summarized evidence, several practical implications for prevention and early intervention can be proposed. Maintaining spinal health requires an integrative approach that encompasses posture, nutrition, physical activity, and psychosocial context. Excessive screen time, sedentary behavior, inadequate diets, and obesity often co-occur and may act synergistically, forming a self-reinforcing network of risk factors. Addressing these issues requires comprehensive preventive programs and a shift in clinical awareness, recognizing that spinal pain and dysfunction in youth should no longer be regarded as rare or incidental findings. Increased recognition of early functional and structural changes, coupled with educational and public health initiatives, will be essential to protect spinal health in future generations.

## Figures and Tables

**Table 1:** Anatomical features of the immature spine that lead to flexion-extension injury sensitivity. Source: Prepared by the authors (2025) in reference to [13,14,17,18].

Anatomical Feature	Contribution to Injury Sensitivity
Proportionally greater weight of the head to the thorax	Increases momentum and strain on the cervical spine during rapid movements
Not fully developed neuro-muscular control of the head-neck-thorax	Reduces the ability to stabilize and protect the spine during dynamic loading
Immature paraspinous musculature	Limits active support and shock absorption along the spinal column
Wedge-shaped vertebral bodies	Alters load distribution and increases the risk of anterior compression injuries
More elastic ligaments	Allows excessive motion, increasing the risk of hyperflexion or hyperextension
Horizontally oriented facet joints	Less effective in limiting excessive motion, especially in extension
More elastic/hydrated discs and annulus fibrosus	Greater deformation under load, which may lead to instability or internal disc injury

**Table 2:** Screen exposure, sedentary behavior, and musculoskeletal outcomes in children and adolescents.

First Author	Year	Study Design	Population	Main Musculoskeletal or Behavioral Findings	Ref.
Rideout V. et al.	2022	Survey	U.S. teenagers	COVID-19 pandemic increased screen time from 4 h 44 min to 5 h 33 min (tweens) and 7 h 22 min to 8 h 39 min (teens)	[45]
Slater S. O. et al.	2025	Cross-sectional	384,591 15-year-olds from 55 countries	Age of first use of a digital device: 70.92% start before age 10.	[46]
Frata B. et al.	2021	Cross-sectional	237 children aged 3–5 years	Predictors for screen time: averaged—4 h/day; positive association between parental and child screen use; familial habits significantly influence exposure	[47]
Hantal A. O. et al.	2024	Case-control	94 patients aged 10–16 with disc pathology	LANDMARK STUDY: The findings from the study group suggest that prolonged use of digital devices and physical inactivity are significant contributors to intervertebral disc degeneration and hernia formation.	[48]
Vagka E. et al.	2023	Cross-sectional	1408 young adults aged 18–25 years.	Prevalence and correlations of nomophobia—99.9% of participants exhibited any level of nomophobia.	[49]
Jahrami H. et al.	2023	Meta-analysis	52 studies, 47,400 participants from 20 countries	Overall prevalence of nomophobia: mild 25%, moderate 50%, severe 20%).	[50]
Dong N. et al.	2025	Longitudinal observation	4557 Adolescents	Problematic screen media use was examined with respect to health outcomes, with psychological hazards as the primary focus.	[51]
Faeze S. et al.	2022	Cross-sectional	80 College students	The angles while using a smartphone differed significantly across positions.	[52]
In T.S. et al.	2021	Biomechanical study	50 Adolescents	Spinal and pelvic malalignment during prolonged sitting with digital devices.	[53]
Oh K.H. et al.	2025	The National Health and Nutrition Examination Survey (2011–2014)	1449 Adolescent	Association of sedentary behavior with musculoskeletal weakness.	[56]
Guthold R. et al.	2020	Global survey	1.6 million students, 298 surveys, 146 countries (2001–2016)	Global trends in insufficient physical activity among adolescents: >80% do not meet WHO recommendations; interventions are particularly important for girls.	[58]
Joergensen A. C. et al.	2021	Cohort study	45,555 pre-adolescents	Duration of screen time is associated with a greater risk of severe spinal pain in girls and boys.	[59]
Seyedahmadi M. et al.	2025	Descriptive-correlational study	200 male students aged 13 to 15 years	Relationship between mobile device use and malalignment of sagittal curvatures.	[60]

Summary: Digital device use begins early, with 70.92% of children initiating use before the age of 10, and average daily exposure frequently exceeding 5–8 h among adolescents. Prolonged screen time has been associated with spinal pain and, in case–control data, with intervertebral disc pathology in adolescents. High prevalence of problematic device-related behaviors (including nomophobia) has also been reported. Additionally, more than 80% of adolescents worldwide do not meet the WHO physical activity recommendations.

## Data Availability

No new data were created or analyzed in this study. Data sharing is not applicable to this article.
